# The lncRNAs *PCGEM1* and *PRNCR1* are not implicated in castration resistant prostate cancer

**DOI:** 10.18632/oncotarget.1846

**Published:** 2014-03-23

**Authors:** John R. Prensner, Anirban Sahu, Matthew K. Iyer, Rohit Malik, Benjamin Chandler, Irfan A. Asangani, Anton Poliakov, Ismael A. Vergara, Mohammed Alshalalfa, Robert B. Jenkins, Elai Davicioni, Felix Y. Feng, Arul M. Chinnaiyan

**Affiliations:** ^1^ Michigan Center for Translational Pathology, University of Michigan, Ann Arbor, Michigan USA; ^2^ Department of Computational Medicine and Bioinformatics, Ann Arbor, Michigan USA; ^3^ GenomeDx Biosciences Inc., Vancouver, British Columbia, Canada; ^4^ Department of Laboratory Medicine and Pathology, Mayo Clinic, Rochester, Minnesota USA; ^5^ Department of Radiation Oncology, University of Michigan, Ann Arbor, Michigan USA; ^6^ Department of Pathology, University of Michigan, Ann Arbor, Michigan USA; ^7^ Comprehensive Cancer Center, University of Michigan, Ann Arbor, Michigan USA; ^8^ Howard Hughes Medical Institute, University of Michigan, Ann Arbor, Michigan USA

**Keywords:** prostate cancer, long noncoding RNA, androgen receptor

## Abstract

Long noncoding RNAs (lncRNAs) are increasingly implicated in cancer biology, contributing to essential cancer cell functions such as proliferation, invasion, and metastasis. In prostate cancer, several lncRNAs have been nominated as critical actors in disease pathogenesis. Among these, expression of *PCGEM1* and *PRNCR1* has been identified as a possible component in disease progression through the coordination of androgen receptor (AR) signaling (Yang et al., Nature 2013, see ref. [1]). However, concerns regarding the robustness of these findings have been suggested. Here, we sought to evaluate whether *PCGEM1* and *PRNCR1* are associated with prostate cancer. Through a comprehensive analysis of RNA-sequencing data (RNA-seq), we find evidence that *PCGEM1* but not *PRNCR1* is associated with prostate cancer. We employ a large cohort of >230 high-risk prostate cancer patients with long-term outcomes data to show that, in contrast to prior reports, neither gene is associated with poor patient outcomes. We further observe no evidence that *PCGEM1* nor *PRNCR1* interact with AR, and neither gene is a component of AR signaling. Thus, we conclusively demonstrate that *PCGEM1* and *PRNCR1* are not prognostic lncRNAs in prostate cancer and we refute suggestions that these lncRNAs interact in AR signaling.

## INTRODUCTION

Long noncoding RNAs (lncRNAs) have emerged as a critical element in cell biology, contributing to a wide variety of cellular behaviors and functions [2]. In cancer, lncRNAs have been the subject of much research during the past five years. Notably, lncRNAs are known to coordinate aggressive phenotypes of several common tumors, including breast cancer and prostate cancer [3, 4]. Large profiling studies have suggested that upwards of 10,000 lncRNAs may exist in the human genome [5]; yet only a fraction of these entities have been characterized. Thus, the identity and function of lncRNAs in cancer remains largely unknown.

In prostate cancer, several lncRNAs, including *PCA3* and *PCAT-1*, have been shown to be upregulated in patients with cancer [6-9]. Recently, two lncRNAs, *PCGEM1* and *PRNCR1*, have been suggested in prostate cancer to act as mediators of castration-resistance disease by binding, in a direct and sequential fashion, to the androgen receptor (AR), causing ligand-independent activation of its gene expression programs [1]. While *PCGEM1* has been observed in prostate cancer previously [6, 10], *PRNCR1* is a poorly characterized transcript, and we were concerned that *PRNCR1* had been nominated by previous global profiling studies of prostate cancers [7, 11-14].

We therefore sought to investigate *PRNCR1* and *PCGEM1* in prostate cancer. In specific, we sought to reproduce three core observations suggested by Yang et al published in *Nature* (see [1]) and include: 1) that *PRNCR1* and *PCGEM1* are highly overexpressed in aggressive forms of prostate cancer, 2) that these two lncRNAs bind to AR under ligand-stimulated conditions, and 3) that the coordination of *PRNCR1* and *PCGEM1* interact with AR via specific post-translational modifications of the AR protein. Here, we report that none of these three findings is fully reproducible.

**Figure 1:**
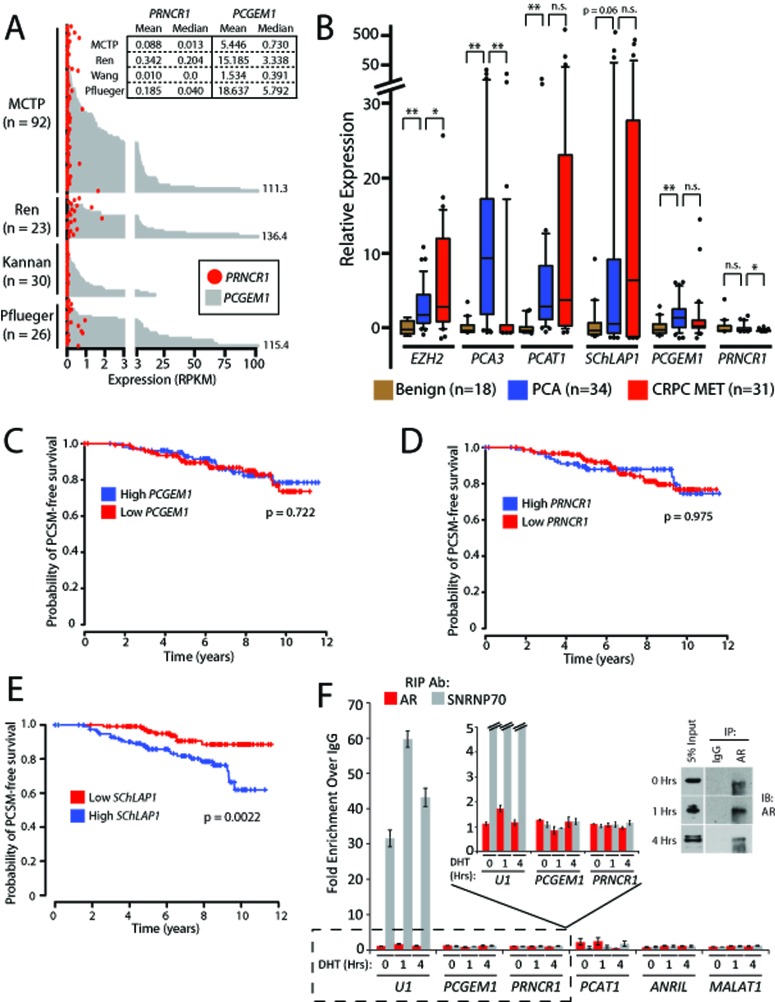
PCGEM1 and PRNCR1 are not associated with prostate cancer progression and do not bind the androgen receptor (A) Plot showing *PCGEM1* (grey bars) and *PRNCR1* (red circles) expression levels (Reads per Kilobase per Million Reads, or RPKM) across 171 samples from four RNA-Seq studies of prostate cancer: Michigan Center for Translational Pathology (MCTP, internal data and dbGAP, phs000443.v1.p1), Ren et al. [13] (EGA, ERP00550), Kannan et al. [14] (GEO, GSE22260), and Pflueger et al. [12] (dbGAP, phs000310.v1.p1). Inset box shows descriptive statistics for each study. (B) Quantitative PCR for *PCGEM1* and *PRNCR1* in a cohort of prostate cancer tissues, benign (n = 18), localized cancer (n =34), metastatic cancer (n = 31). An asterisk (*) indicates p < 0.05. Two asterisks (**) indicate p < 0.01. n.s. = non-significant. P values were determined by a two-tailed Student's t-test. Data for *SChLAP1* is obtained and re-analyzed from a prior publication (ref. [4]). (C) *PCGEM1* expression does not predict for prostate cancer-specific mortality (PCSM). (D) *PRNCR1* expression does not predict for PCSM. (E) High *SChLAP1* expression is a powerful predictor of PCSM (p = 0.0022). Data in (E) is reproduced from a prior publication (ref. [4]). P values in (C-E) are determined using a log-rank test. (F) RNA- immunoprecipitation (RIP) for AR following stimulation of LNCaP cells with 100nM DHT does not show binding of *PRNCR1* or *PCGEM1* to AR. *U1* binding to SNRNP70 is used as a positive control. *PCAT-1*, *ANRIL*, and *MALAT1* serve as negative controls. Inset: Western blot confirmation of AR protein pull-down by the immunoprecipitation assays. Error bars represent S.E.M.

First, we asked whether *PCGEM1* and *PRNCR1* are highly overexpressed in aggressive prostate cancer, as suggested by others (see [1, 15]). Indeed, while some have argued that these lncRNAs are critical in castration-resistant prostate cancer [1], there has been no study that evaluated the expression of these lncRNAs in tissue samples from human castrate-resistant prostate cancers (CRPC). To evaluate these lncRNAs in more detail, we first assessed their expression levels in 171 human prostatic tissues using RNA sequencing data aggregated from four independent studies of prostate cancer, including our own internal datasets [1, 12-14] (Fig. 1A). Whereas we found robust expression of *PCGEM1* in a subset of prostate tissues (RPKM >1 in 82 samples; RPKM >10 in 27 samples), we observed scant levels of *PRNCR1* in all samples (RPKM >1 in only 3 samples; RPKM >10 in 0 samples) (Supplementary Table 1). This does not lend confidence to *PRNCR1* as a significant entity in this disease. For comparison, we used the prostate cancer lncRNA *SChLAP1* as a positive control. We found extreme overexpression of *SChLAP1* in samples from all datasets (RPKM >1 in 69 samples; RPKM >10 in 26 samples) (Supplementary Fig. 1). To rule out the possibility that *PRNCR1* was a non-poly-adenylated RNA, we verified experimentally that *PRNCR1* was observed in the poly-A fraction of RNA that was used to generate the RNA-seq data (Supplementary Fig. 2).

Given the low support for *PRNCR1* in the RNA-seq data, we next confirmed these findings using quantitative PCR (qPCR) in a large set of prostate cancer tissues including 34 PCAs and 31 CRPC tumors as well as 18 benign adjacent tissues. As shown in Fig. 1B, *PCGEM1* is upregulated in clinically localized cancer, confirming the known literature [6, 10]; however *PRNCR1* expression does not demonstrate a convincing association with prostate cancer. We found a borderline decrease in *PRNCR1* expression in metastatic castrate-resistant cancer (p = 0.047, Student's t-test). We used *PCAT1*, *EZH2*, and *SChLAP1* as control genes, all of which have elevated expression in prostate cancer metastases. Conversely, we used *PCA3* as a control gene that is known to be upregulated in localized prostate cancer but not metastatic prostate cancer. Finally, while *PCGEM1* is upregulated in cancer patients from matched tumor/benign samples, *PRNCR1* does not convincingly exhibit this pattern of upregulation (Supplementary Fig. 3).

Next, an independent analysis of 235 high-risk prostate cancer tissues demonstrated that neither *PCGEM1* nor *PRNCR1* is associated with aggressive prostate cancer, and neither lncRNA stratifies prostate cancer-specific mortality (Fig. 1C,D and Supplementary Tables 2,3). An analysis of intermediate endpoints such as biochemical recurrence and progression to metastatic disease demonstrated a trend for *PCGEM1* and *PRNCR1* to be associated with less aggressive disease and favorable outcomes (Supplementary Fig. 4), which contradicts previous claims that these lncRNAs are involved in an aggressive patient clinical course [1, 15]. Using an independent validation cohort of tissues we verified that neither *PCGEM1* nor *PRNCR1* is associated with aggressive prostate cancer (Supplementary Table 2). By contrast, we have used these datasets to confirm the prognostic utility of the lncRNA *SChLAP1* in prostate cancer, and high expression of *SChLAP1* is a powerful predictor for poor patient survival (Fig. 1E) [4].

Next, we examined whether *PCGEM1* and *PRNCR1* interacted with AR. We performed RNA-IP (RIP) assays using two independent AR antibodies, including the same antibody that was previously used to show an interaction between these lncRNAs and AR [1]. In accordance with the published literature, we performed a time-series of RIP experiments following AR stimulation, because prior data suggests that these lncRNAs bind AR from 1-2 hours after AR stimulation but not at 4 hours post-stimulation [1]. In our RIP experiments, we could not confirm that AR binds to *PCGEM1* or *PRNCR1* at either 1 hour or 4 hours post-stimulation with DHT (Fig. 1F and Supplementary Fig. 5). Similarly, in cells grown at steady-state, we used a second AR antibody and did not observe binding between AR and *PCGEM1* or *PRNCR1* (Supplementary Fig. 6). DHT-stimulated cells also demonstrated no induction in *PCGEM1* or *PRNCR1* expression (Supplementary Fig. 7). These results imply that *PCGEM1* and *PRNCR1* are not AR-interacting lncRNAs.

Finally, earlier data propose that *PCGEM1* and *PRNCR1* interact with AR via specific post-translational modifications (PTMs), specifically K349 methylation (K349Me) for *PCGEM1* and K631/K634 acetylation (K631Ac/K634Ac) for *PRNCR1* [1]. To search for these PTMs, we independently performed mass spectrometry for AR in the LNCaP cell line, achieving 95% coverage of all possible tryptic peptides. We were unable to confirm that these PTMs (K349Me, K631Ac, or K634Ac) are present on AR (Supplementary Fig. 8 and Supplementary Table 4). To examine this discrepancy further, we re-analyzed prior AR MS data (found in [1]). Although this MS dataset was obtained with a trypsin digestion to prepare samples for MS, we found no fully tryptic peptides supporting the nomination of K349Me, K631Ac, or K634Ac (Supplementary Fig. 8). In fact, in the MS data for these PTMs in ref. [1], almost all peptides harboring these PTMs are non-tryptic, which are generally considered to be analysis artifacts since true non-tryptic peptides are exceedingly rare following a trypsin digestion [16-18] (Supplementary Discussion). Non-tryptic peptides are also associated with a high false-discovery rate [19]. All peptides nominating the K349Me, K631Ac, or K634Ac PTMs in ref. [1] also had multiple additional PTMs that were nominated, indicating non-specificity. These included extraordinarily rare and unusual PTMs such as oxidated lysine and deamidated asparagine, which suggest technical artifacts given the negligible likelihood of multiple rare and unusual PTMs occurring on true non-trypic peptides. The statistical confidence for these non-tryptic peptides is <5%, whereas the corresponding fully tryptic peptides for these amino acid residues had statistical confidences >90%.

In summary, we have been unable to show a convincing role for *PCGEM1* or *PRNCR1* in aggressive prostate cancer or AR signaling. First, our data analysis of numerous human prostate cancer tissues from multiple independent laboratories indicates that neither *PCGEM1* nor *PRNCR1* are associated with castration-resistant prostate cancer. Second, we were unable to verify that *PCGEM1* and *PRNCR1* bind to the androgen receptor. Lastly, we are unconvinced that the K349Me, K631Ac, or K634Ac AR PTMs represent a plausible mechanism for interaction between AR and *PCGEM1* and *PRNCR1*. While our results challenge the notion that *PCGEM1* and *PRNCR1* play a causal role in prostate cancer, we regard lncRNAs as an emerging field of study in cancer [3, 6, 20, 21] and we are encouraged by the interest in lncRNAs in prostate cancer.

## METHODS

Prostate tissues were obtained from the radical prostatectomy series and Rapid Autopsy Program at the University of Michigan tissue core. All tissue samples were collected with informed consent under an Institutional Review Board (IRB) approved protocol at the University of Michigan. Outcomes analyses were performed on a cohort of Mayo Clinic prostate cancer radical prostatectomy samples obtained under an IRB-approved protocol as described previously. Cell lines were maintained according to standard conditions. For RIP experiments, cells were deprived of androgen for 48 hours prior to stimulation with 100nM DHT. RIP experiments were performed as previously described [1, 4]. Bioinformatics analyses utilized publicly available RNA-Seq data. Please see Supplementary Methods for details.

## SUPPLEMENTARY FIGURES AND TABLES




