# Supplementary material for: The lncRNAs PCGEM1 and PRNCR1 are not implicated in castration resistant prostate cancer
Source: Oncotarget. 2014 Mar 23;5(6):1434–8. doi: 10.18632/oncotarget.1846 (PMC4039221; doi:10.18632/oncotarget.1846)
Supplement: Supplementary file 1 [file oncotarget-05-1434-s001.pdf]

Supplementary Materials

The lncRNAs *PCGEM1* and *PRNCR1* are not implicated in castration resistant prostate cancer

John R. Prensner[1,9], Anirban Sahu[1,9], Matthew K. Iyer[1,2,9], Rohit Malik[1,9], Benjamin Chandler[1], Irfan A. Asangani[1], Anton Poliakov[1], Ismael A. Vergara[3], Mohammed Alshalalfa[3], Robert B. Jenkins[4], Elai Davicioni[3], Felix Y. Feng[1,5,7], Arul M. Chinnaiyan[1,2,6,7,8]

[1]Michigan Center for Translational Pathology, University of Michigan, Ann Arbor, Michigan USA.
[2]Department of Computational Medicine and Bioinformatics, Ann Arbor, Michigan USA.
[3]GenomeDx Biosciences Inc., Vancouver, British Columbia, Canada.
[4]Department of Laboratory Medicine and Pathology, Mayo Clinic, Rochester, Minnesota USA.
[5]Department of Radiation Oncology, University of Michigan, Ann Arbor, Michigan USA.
[6]Department of Pathology, University of Michigan, Ann Arbor, Michigan USA.
[7]Comprehensive Cancer Center, University of Michigan, Ann Arbor, Michigan USA.
[8]Howard Hughes Medical Institute, University of Michigan, Ann Arbor, Michigan USA.
[9]These authors contributed equally

Key words: prostate cancer, long noncoding RNA, androgen receptor

Address correspondence to:
Arul M. Chinnaiyan, M.D. Ph.D.
Investigator, Howard Hughes Medical Institute
Comprehensive Cancer Center
University of Michigan Medical School
1400 E. Medical Center Dr. 5316 CCGC 5940
Ann Arbor, MI 48109-5940
arul@med.umich.edu

Methods
Cell lines
All cell lines were obtained from the American Type Culture Collection (Manassas, VA). Cell lines were maintained using standard media and conditions. Specifically, VCaP cells were maintained in DMEM (Invitrogen) plus 10% fetal bovine serum (FBS) plus 1% penicillin-streptomycin. LNCaP cells were maintained in RPMI 1640 (Invitrogen) plus 10% FBS and 1% penicillin-streptomycin.

All cell lines were grown at 37°C in a 5% CO_2 cell culture incubator. All cell lines were genotyped for identity at the University of Michigan Sequencing Core.

Tissue Samples
Prostate tissues were obtained from the radical prostatectomy series and Rapid Autopsy Program at the University of Michigan tissue core [1]. These programs are part of the University of Michigan Prostate Cancer Specialized Program Of Research Excellence (S.P.O.R.E.). All tissue samples were collected with informed consent under an Institutional Review Board (IRB) approved protocol at the University of Michigan. (SPORE in Prostate Cancer (Tissue/Serum/Urine) Bank Institutional Review Board # 1994-0481).

RNA isolation and cDNA synthesis
Total RNA was isolated using Trizol and an RNeasy Kit (Invitrogen) with DNase I digestion according to the manufacturer's instructions. RNA integrity was verified on an Agilent Bioanalyzer 2100 (Agilent Technologies, Palo Alto, CA). cDNA was synthesized from total RNA using Superscript III (Invitrogen) and random primers (Invitrogen).

For RNA fractionation experiments, poly-adenylated RNA was isolated using oligo-dT(20) beads as previously described for mRNA-Seq protocols (Illumina). Poly-A RNA was hybridized to the oligo-dT(20), washed with standard salt washes as described in Illumina mRNA-seq protocols, and eluted in 10mM Tris. The non-captured RNA supernatant was used for non-poly-A RNA. RNA was precipitated with ethanol using standard protocols and converted into cDNA using Superscript III as above.

Quantitative Real-time PCR
Quantitative Real-time PCR (qPCR) was performed using Power SYBR Green Mastermix (Applied Biosystems, Foster City, CA) on an Applied Biosystems 7900HT Real-Time PCR System. All oligonucleotide primers were obtained from Integrated DNA Technologies (Coralville, IA) and are listed in **Supplementary Table 5.** The housekeeping genes, *GAPDH, HMBS,* and *ACTB*, were used as loading controls. Fold changes were calculated relative to housekeeping genes and normalized to the median value of the benign samples.

Immunoblot Analysis
Cells were lysed in RIPA lysis buffer (Sigma, St. Louis, MO) supplemented with HALT protease inhibitor (Fisher). Western blotting analysis was performed with standard

protocols using Polyvinylidene Difluoride membrane (GE Healthcare, Piscataway, NJ) and the signals visualized by enhanced chemiluminescence system as described by the manufacturer (GE Healthcare).

Protein lysates were boiled in sample buffer, and 10 ug protein was loaded onto a SDS-PAGE gel and run for separation of proteins. Proteins were transferred onto Polyvinylidene Difluoride membrane (GE Healthcare) and blocked for 90 minutes in blocking buffer (5% milk, 0.1% Tween, Tri-buffered saline (TBS-T)). Membranes were incubated overnight at 4C with primary antibody. Following 3 washes with TBS-T, and one wash with TBS, the blot was incubated with horseradish peroxidase-conjugated secondary antibody and the signals visualized by enhanced chemiluminescence system as described by the manufacturer (GE Healthcare).

Primary antibodies used were:
ACTB (1:5000, Sigma, A5316, mouse)
AR (1:1000, Millipore, 06-680, rabbit)
AR (1:1000, Santa Cruz, sc-7305, mouse)

DHT Stimulation Assay:
LNCaP cells were serum starved in RPMI containing 5% charcoal stripped media for 48 hours and then stimulated with 10nM DHT for various time points. Cells were lyses in QIAzol Lysis reagent (Qiagen Cat. No. 79306) and RNA was extracted using miRNeasy Mini Kit (Qiagen Cat. No. 217004). cDNA was synthesized using SuperScript® III First Strand Synthesis Kit and Random primers. qRT-PCR was carried out on the ABI7900 HT Fast Real time system (Applied Biosystems) using gene-specific primer. qRT-PCR data were analyzed using the relative quantification method and plotted as average fold-change compared with the control. *GAPDH* and *ACTB* were used as controls.

RNA immunoprecipitation
LNCaP cells were serum starved in RPMI containing 5% charcoal stripped media for 48 hours and then stimulated with 100 nM DHT for various time points. Cells were trypsonized and washed once with cold PBS. 10^7 cells were pelleted by centrifugation and nuclei were obtained using NE-PER nuclear extraction kit (Thermo Scientific). Nuclear fraction lysates were used as an input in the RIP assays.

RIP assays were performed using a Millipore EZ-Magna RIP RNA-Binding Protein Immunoprecipitation kit (Millipore, #17-701) according to the manufacturer's instructions. Conditions for RIP were similar to those previously described [2, 3]. RIP-PCR was performed as qPCR, as described above, using total RNA as input controls. 1:150th of RIP RNA product was used per PCR reaction. Antibodies used for RIP were polyclonal IgG (Millipore, PP64), SNRNP70 (Millipore, CS203216), AR (Millipore, 06-680), and AR (Santa Cruz, sc-7305) using 5 ug of antibody per RIP reaction.

Mass Spectrometry
IP beads were washed and all bound proteins were eluted by incubating for 5 min at 85°C in SDS-PAGE loading buffer containing 10 mM DTT. The proteins alkylated by

iodoacetomide and separated on 4-12% SDS-PAGE gel and stained with Coomassie R-250. The area of gel corresponding to MW from 90 to 110 (where AR should be located) was excised and digested with trypsin. The resulting peptides were loaded on C18 nano column (10 cm long, 75 µm internal diameter) and eluted with a gradient of acetonitrile into Velos Pro mass spectrometer operated in data-dependent mode. The resulting files were transferred into .mgf format with MSConvert converter and interrogated against SwissProt human database version 15 using MASCOT search engine. The search parameters were as follows: trypsin, 2 missed cleavages allowed, fixed modification (cysteine carbamidomethylation), variable modifications (protein N-terminal acetylation, lysine acetylation and methionine oxidation), precursor mass tolerance 1 Da, fragment mass tolerance 0.5 Da.

This analysis has been repeated 3 times and all MS results were merged. The combined MASCOT search result was filtered to obtain peptide FDR (false discovery rate) of less than 1% by adjusting significance threshold and ions score.

Statistical analyses for experimental studies
All data are presented as means ± S.E.M. Statistical analyses shown in figures represent two-tailed t-tests, as indicated. Statistical considerations for mass spectrometry are detailed above.

<u>Bioinformatics Analysis</u>
We obtained paired-end RNA-seq data from four prostate cancer datasets: Michigan Center for Translational Pathology [4] (MCTP, dbGAP phs00443.v.p1), Ren et al. [5] (EGA, ERP00550), Kannan et al. [6] (GEO, GSE22260), and Pflueger et al. [7] (dbGAP, phs000310.v1.p1). The sequences from each RNA-seq experiment were aligned using Tophat version 2.0.7 [8]. To assess expression levels we quantified unique read alignments spanning contiguous predefined transcript intervals. This approach is akin to measuring hybridization to predefined microarray probes, or by quantifying transcript cDNA with predetermined qPCR primers. For reads partially overlapping an interval we only added the fraction of overlapping bases of the read to the final count. We chose ~1kb intervals from each transcript for quantification. For *PRNCR1* we assessed reads overlapping the interval chr8:128,095,130-128,096,130 (UCSC genome version hg19), which encompasses the qPCR amplicon assayed by Yang et al. For *PCGEM1* we counted reads overlapping the interval chr2:193,640,424-193,641,433 on the transcript's 3' UTR. Read counts were normalized to Reads Per Kilobase Per Million Reads (RPKM) using the length of the probe interval and the total number of unique read alignments in each library.

Plot showing PCGEM1 (grey bars) and PRNCR1 (red circles) expression levels (Reads per Kilobase per Million Reads, or RPKM) across 171 samples from four RNA-Seq studies of prostate cancer: Michigan Center for Translational Pathology (MCTP, internal data and dbGAP, phs000443.v1.p1),

Mayo Clinic Cohort Analyses

Clinical Cohort

The clinical study design and patient cohort characteristics are described elsewhere [2, 9]. Patients were from a high-risk cohort defined as pre-operative PSA >20 ng/ml, pathological Gleason score 8-10, seminal vesicle invasion (SVI), or GPSM score >=10 [10]. Patients were evaluated for biochemical recurrence of PSA (BCR), clinical progression to metastatic disease by imaging (CP), and prostate-cancer specific mortality (PCSM) as described previously.

RNA Extraction and Microarray Hybridization

Tissue from formalin-fixed paraffin embedded (FFPE) samples of human prostate adenocarcinoma prostatectomies were macrodissected using guide H&E stains. The index lesion was considered the dominant lesion by size. Total RNA was extracted and purified using a modified protocol for the commercially available RNeasy FFPE nucleic acid extraction kit (Qiagen Inc., Valencia, CA). Purified total RNA was subjected to whole-transcriptome amplification using the WT-Ovation FFPE system according to the manufacturer's recommendation with minor modifications (NuGen, San Carlos, CA). For the validation only the Ovation® FFPE WTA System was used. Amplified products were fragmented and labelled using the Encore™ Biotin Module (NuGen, San Carlos, CA) and hybridized to Affymetrix Human Exon (HuEx) 1.0 ST GeneChips following manufacturer's recommendations (Affymetrix, Santa Clara, CA).

Expression Analysis

The normalization and summarization of the microarray samples was done with the frozen Robust Multiarray Average (fRMA) algorithm using custom frozen vectors. These custom vectors were created using the vector creation methods as described previously [11]. Quantile normalization and robust weighted average methods were used for normalization and summarization, respectively, as implemented in fRMA.

For PCGEM1, 4 probes matching to this gene were averaged to generate a gene-level representation of gene expression. For PRNCR1, only one probe was found to map to this sequence and was used for gene expression analyses. Expression analyses were performed as previously described [2].

Statistical Analysis

Mayo clinic samples were analyzed using Kaplan-Meier curves. P values were determined using a log-rank test.

Supplementary Discussion

Upon close inspection of the data presented in the manuscript by Yang et al. [3], we have discovered that none of the peptides where AR modifications (Supplementary Fig. 4e in Yang et al.) were indicated are fully tryptic (**Supplementary Fig. 8A**). This is surprising since trypsin has been used for digestion of the proteins and none of these modifications were detected in the overlapping tryptic peptides. Abundance of fully non-tryptic peptides is usually extremely low and their identification is associated with high false discovery rate [12]. Accordingly, all peptides listed in the Supplementary Fig. 4e (in Yang et al.) have very low confidence levels (as reported by ProteinPilot [13]) and cannot be counted as valid.

Yang et al. have concluded that the lack of interaction of *PCGEM1* with AR K349R mutant is a proof of K349 methylation. Likewise, the lack of interaction of AR K631/634R mutant with *PRNCR1* and *PCGEM1* is considered a proof of K631 and K634 acetylation. However, this proof is recursive and the data only indicate that K349, K631 and K634 might be important for the above mentioned interactions and that the effect could have been obtained even if the lysines were not modified.

With respect to K349 methylation specifically, modification of lysine by either methyl or acetyl group prevents cleavage at this residue by trypsin due to significantly perturbed geometry and electrostatic properties of the side chain. Accordingly, a non-tryptic peptide T-LSLYKSGALDE-A, where methylation of K349 has been suggested (Supplementary Fig. 4e in Yang et al.), has a missed cleavage at K349. This peptide has a very low confidence of 0.57, suggesting that this identification is unlikely to be valid. Additionally, in the Supplementary Table 2 (in Yang et al.) a fully tryptic peptide with the sequence K-SGALDEAAAYQSR-D is listed with a confidence of 99, suggesting that this is a valid identification. This peptide overlaps with T-LSLYKSGALDE-A (see **Supplementary Fig. 8B**) and is a result of a cleavage between K349 and S350. The fact that a peptide

bond can be cleaved after K349 directly indicates that K349 is not modified, since K349-S350 peptide bond would have not been cleaved if K349 was methylated.

In order to check the PTM status of the above-mentioned AR residues, we have performed an extensive mass spectrometric analysis of AR from LNCaP cells. We performed three mass spectrometric analyses and have achieved 66% total sequence coverage of AR and 95% coverage if counting all possible tryptic peptides (**Supplementary Fig. 8C** and **Supplementary Table 4**) at a confidence cutoff level of a peptide FDR<1%. Specifically, in the N-terminal domain all lysines were accounted for as they were either a part of one of the detected peptides or were immediately upstream of them (**Supplementary Fig. 8C**), suggesting that they were not modified as trypsin cleavage would not occur on an acetylated or methylated lysines. We have found no evidence for K349 methylation and K631/K634 acetylation and thus cannot confirm the findings presented in the Yang et al. publication.

Of note, we were not able to obtain the original MS files for the Yang et al. manuscript from Dr. Rosenfeld and his research group, despite several email communications from them regarding this matter. We would encourage Dr. Rosenfeld to make his raw files publicly available for the research community to analyze. This can be done using a standard database for the deposition of MS data such as the PRIDE database.

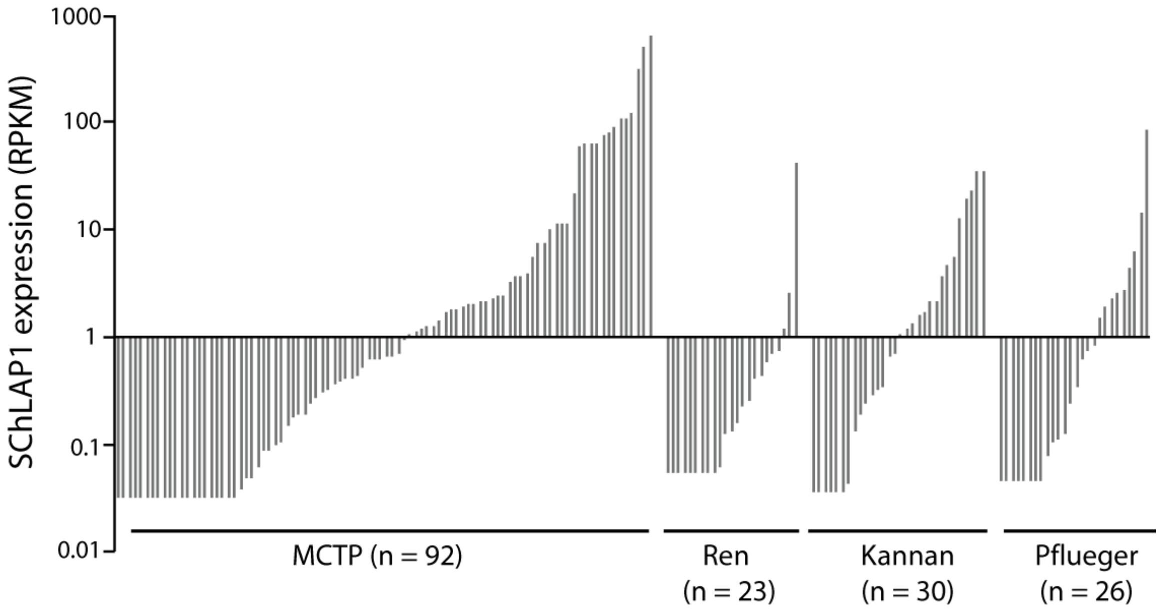

Supplementary Figure 1: *SChLAP1* expression across four RNA-seq datasets. The four RNA-seq datasets used to investigate *PRNCR1* and *PCGEM1* expression were used to measure *SChLAP1* expression. In accordance with previous studies, *SChLAP1* expression shows an outlier phenotype in which a large proportion of samples have no expression and a smaller subset (<50%) have outlier, or extremely high, expression. All datasets have patient samples with outlier expression. Note that the Y axis represents a log10 scale.

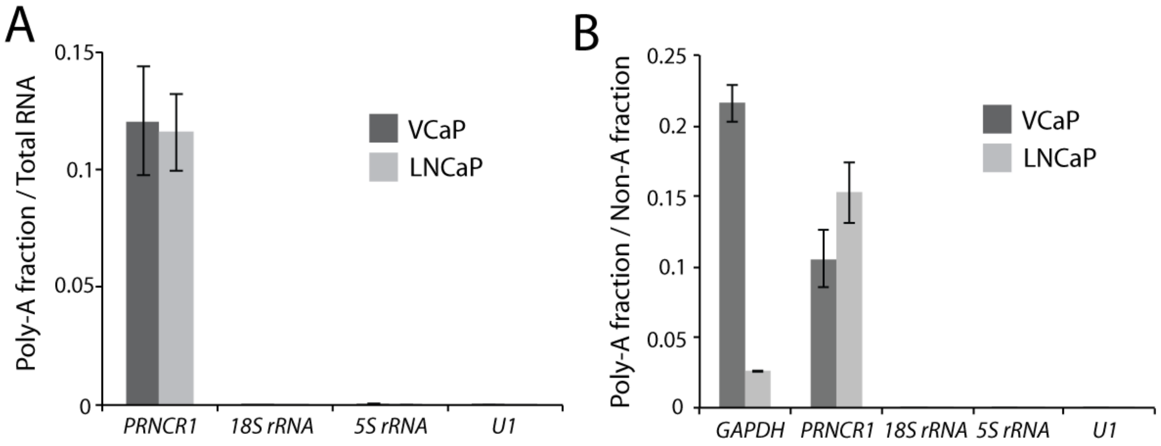

Supplementary Figure 2: PRNCR1 is detectable in poly-A RNA fractions. Total RNA from the LNCAP and VCAP cell lines were fractionated into poly-A and non-poly-A fractions using oligo-dT beads. **(A)** Relative abundance of PRNCR1 in the poly-A fraction compared to total, unfractionated RNA. 18s rRNA, 5s rRNA, and U1 are negative controls that are not poly-adenylated. **(B)** Relative abundance of PRNCR1 in the poly-A fraction compared to the non-poly-A fraction. GAPDH is a positive control. 18s rRNA, 5s rRNA, and U1 are negative controls.

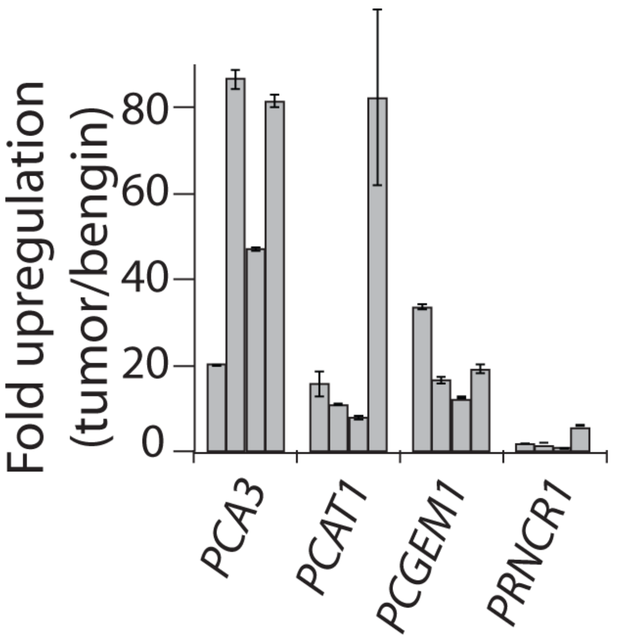

Supplementary Figure 3: Analysis of PCGEM1 and PRNCR1 expression in paired tumor-benign prostate samples. Four patients with matched localized tumor and adjacent benign tissue were evaluated for *PCGEM1* and *PRNCR1* expression by qPCR. *PCA3* and *PCAT1* serve as positive controls. Error bars represent S.E.M.

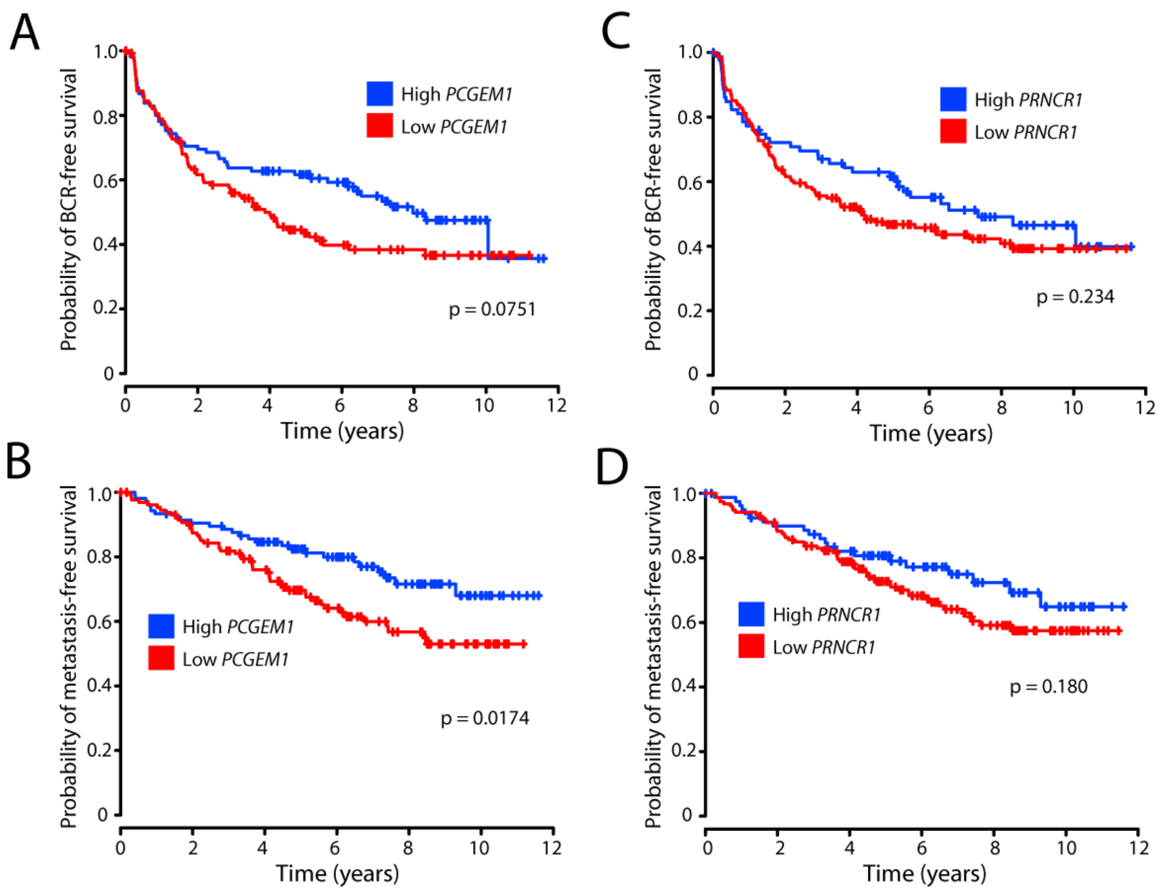

Supplementary Figure 4: PRNCR1 and PCGEM1 are not associated with aggressive prostate cancer. **(A, B)** PCGEM1 is associated with improved outcomes with a lower probability of experiencing biochemical recurrence (BCR) **(A)** or metastasis **(B)** in the Mayo Clinic cohort. **(C,D)** PRNCR1 is not significantly associated with prostate biochemical recurrence **(C)** or metastasis **(D)** in the Mayo Clinic cohort.

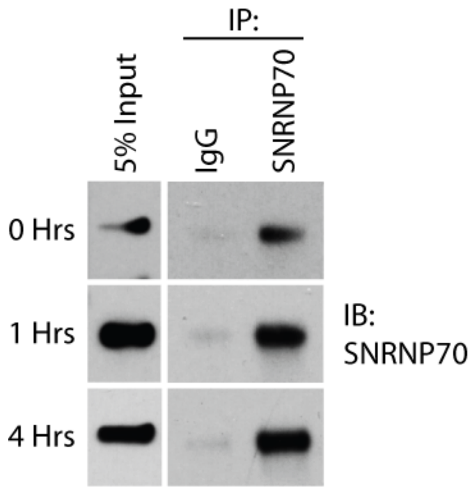

Supplementary Figure 5: Confirmation of SNRNP70 RIP experiments. LNCAP cells were treated with 100nM DHT and cells were harvested at the indicated time-points. RIP was performed for SNRNP70 and protein pull-down was verified by western blotting.

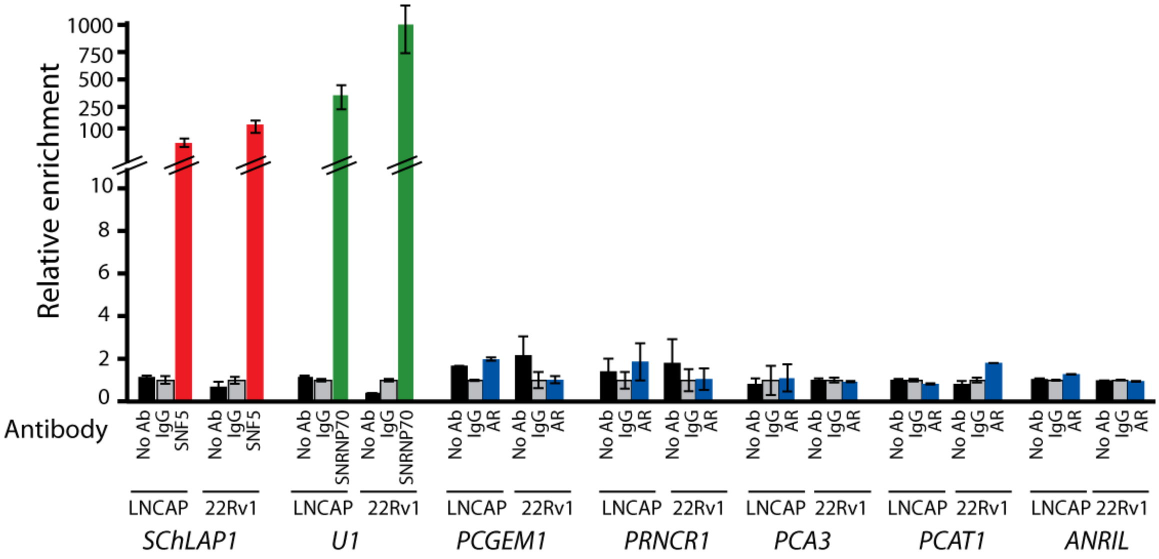

Supplementary Figure 6: PCGEM1 and PRNCR1 do not bind AR under steady-state cell culture conditions. LNCAP or 22Rv1 cells were grown in standard media conditions. RIP experiments were performed for SNF5, SNRNP70, and AR. IgG was used as a negative control. SChLAP1 demonstrates strong binding to SNF5 and U1 demonstrates strong binding to SNRNP70, which serve as positive controls. PCGEM1 and PRNCR1 do not demonstrate binding to AR. PCA3, PCAT1, and ANRIL serve as negative controls.

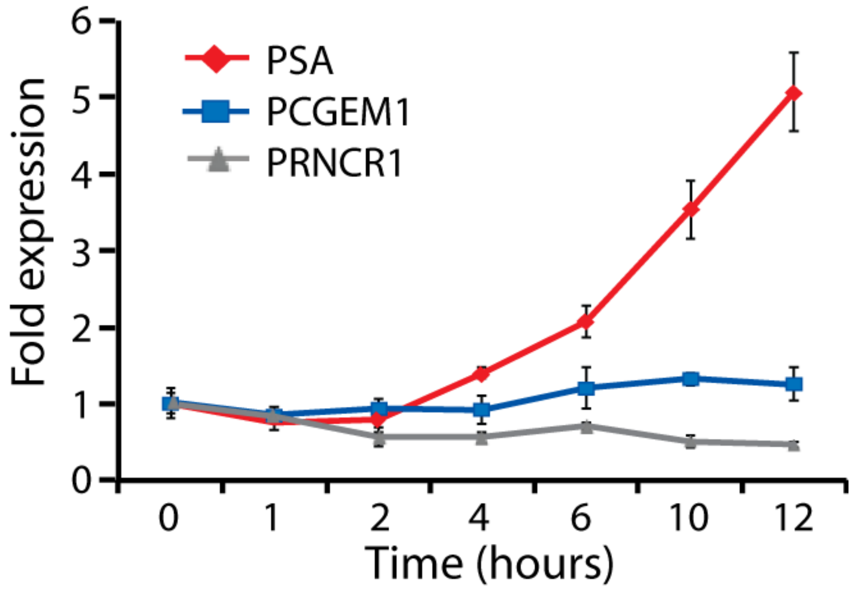

Supplementary Figure 7: PCGEM1 and PRNCR1 are not androgen-regulated genes. Androgen-deprived LNCAP cells were treated with 10nM DHT and RNA was isolated at a variety of time-points. qPCR shows induction of PSA (positive control). PCGEM1 and PRNCR1 demonstrate no change in expression level up stimulation with DHT.

A

MS peptide with adjacent amino acids	Digestion pattern	Missed cleavages	Nominated PTMs	Confidence
V-AASKGLPQQL-P	Non-tryptic	1	Phospho(S)@3 Methyl(K)@4	0.065
K-DNYLGGTSTISDNAKE-L	Semi-tryptic	1	Dehydrated(D)@12 Deamidated(N)@13	46.54
C-KGSLLDDS-A	Non-tryptic	1	Methyl(K)@1 Phospho(S)@3	2.446
Y-TKGLEGESL-G	Non-tryptic	1	Methyl(K)@2 Phospho(S)@8	0.05
T-LSLYKSGALDE-A	Non-tryptic	1	Methyl(K)@5 Phospho(S)@6	0.57
R-KLKKLGNLKLQEEGEA-S	Semi-tryptic	4	Acetyl(K)@1 Oxidation(K)@9	19.29
T-LGARKLKKL-G	Non-tryptic	4	Oxidation(K)@5 Acetyl(K)@8	0.05

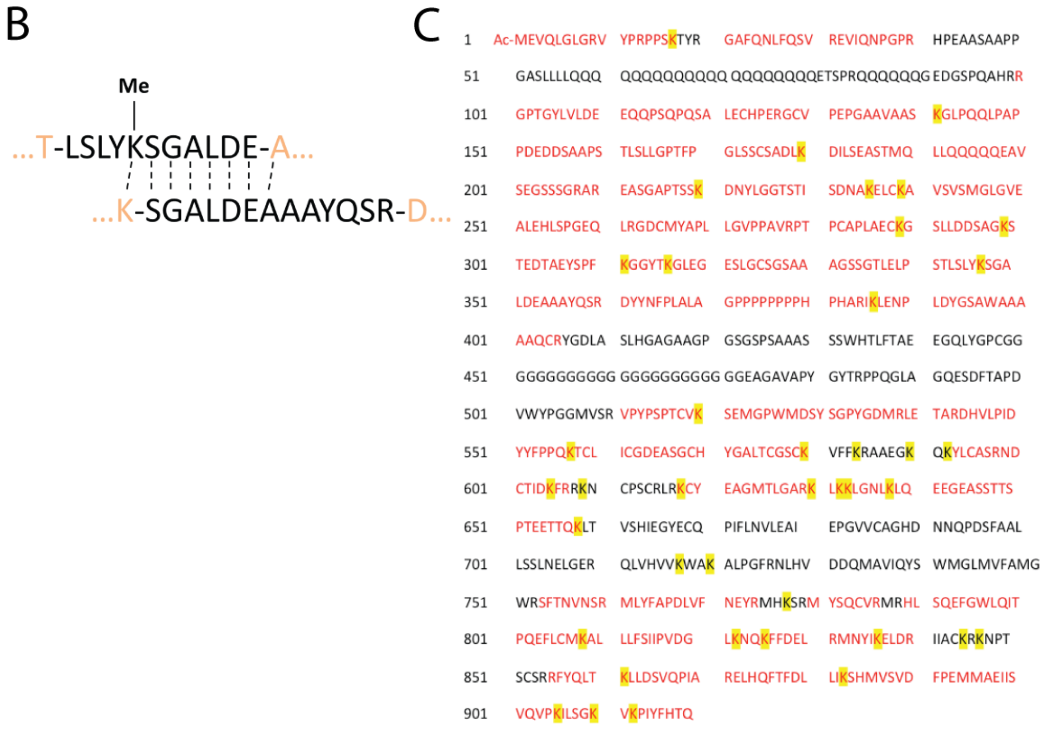

Supplementary Figure 8: AR mass spectrometry data does not support robust post-translational modifications (PTMs) at K349, K631, or K634. (A) A modified version of data from Yang et al. (Supplementary Fig. 4e in that manuscript) listing peptides used to nominate PTMs at K349, K631, and K634. The indicated PTMs are not detected in tryptic peptides overlapping the same residues. Supporting peptides are all non-tryptic and all have at least one missed cleavage. Statistical confidence in these peptides is extremely low. All peptides have numerous PTMs nominated, indicating non-specificity. **(B)** Alignment of the non-tryptic peptide used to nominate K349 methylation (top), which has a statistical confidence of <1%, and the fully tryptic peptide covering that

lysine which does not nominate K349 methylation and has a statistical confidence of 99%. **(C)** Peptide coverage for the University of Michigan AR mass spectrometry. Areas of the AR protein for which peptides were identified are in red. All lysine residues are highlighted in yellow.

Supplementary References

1. Rubin MA, Putzi M, Mucci N, Smith DC, Wojno K, Korenchuk S and Pienta KJ. Rapid ("warm") autopsy study for procurement of metastatic prostate cancer. Clin Cancer Res. 2000; 6(3):1038-1045.
2. Prensner JR, Iyer MK, Sahu A, Asangani IA, Cao Q, Patel L, Vergara IA, Davicioni E, Erho N, Ghadessi M, Jenkins RB, Triche TJ, Malik R, Bedenis R, McGregor N, Ma T, et al. The long noncoding RNA SChLAP1 promotes aggressive prostate cancer and antagonizes the SWI/SNF complex. Nat Genet. 2013.
3. Yang L, Lin C, Jin C, Yang JC, Tanasa B, Li W, Merkurjev D, Ohgi KA, Meng D, Zhang J, Evans CP and Rosenfeld MG. lncRNA-dependent mechanisms of androgen-receptor-regulated gene activation programs. Nature. 2013; 500(7464):598-602.
4. Prensner JR, Iyer MK, Balbin OA, Dhanasekaran SM, Cao Q, Brenner JC, Laxman B, Asangani IA, Grasso CS, Kominsky HD, Cao X, Jing X, Wang X, Siddiqui J, Wei JT, Robinson D, et al. Transcriptome sequencing across a prostate cancer cohort identifies PCAT-1, an unannotated lincRNA implicated in disease progression. Nat Biotechnol. 2011; 29(8):742-749.
5. Ren S, Peng Z, Mao JH, Yu Y, Yin C, Gao X, Cui Z, Zhang J, Yi K, Xu W, Chen C, Wang F, Guo X, Lu J, Yang J, Wei M, et al. RNA-seq analysis of prostate cancer in the Chinese population identifies recurrent gene fusions, cancer-associated long noncoding RNAs and aberrant alternative splicings. Cell Res. 2012; 22(5):806-821.
6. Kannan K, Wang L, Wang J, Ittmann MM, Li W and Yen L. Recurrent chimeric RNAs enriched in human prostate cancer identified by deep sequencing. Proc Natl Acad Sci U S A. 2011; 108(22):9172-9177.
7. Pflueger D, Terry S, Sboner A, Habegger L, Esgueva R, Lin PC, Svensson MA, Kitabayashi N, Moss BJ, MacDonald TY, Cao X, Barrette T, Tewari AK, Chee MS, Chinnaiyan AM, Rickman DS, et al. Discovery of non-ETS gene fusions in human prostate cancer using next-generation RNA sequencing. Genome Res. 2011; 21(1):56-67.
8. Trapnell C, Pachter L and Salzberg SL. TopHat: discovering splice junctions with RNA-Seq. Bioinformatics. 2009; 25(9):1105-1111.
9. Erho N, Crisan A, Vergara IA, Mitra AP, Ghadessi M, Buerki C, Bergstralh EJ, Kollmeyer T, Fink S, Haddad Z, Zimmermann B, Sierocinski T, Ballman KV, Triche TJ, Black PC, Karnes RJ, et al. Discovery and validation of a prostate cancer genomic classifier that predicts early metastasis following radical prostatectomy. PLoS One. 2013; 8(6):e66855.
10. Blute ML, Bergstralh EJ, Iocca A, Scherer B and Zincke H. Use of Gleason score, prostate specific antigen, seminal vesicle and margin status to predict biochemical failure after radical prostatectomy. J Urol. 2001; 165(1):119-125.
11. Vergara IA, Erho N, Triche TJ, Ghadessi M, Crisan A, Sierocinski T, Black PC, Buerki C and Davicioni E. Genomic "Dark Matter" in Prostate Cancer: Exploring the Clinical Utility of ncRNA as Biomarkers. Front Genet. 2012; 3:23.
12. Olsen JV, Ong SE and Mann M. Trypsin cleaves exclusively C-terminal to arginine and lysine residues. Mol Cell Proteomics. 2004; 3(6):608-614.
13. Shilov IV, Seymour SL, Patel AA, Loboda A, Tang WH, Keating SP, Hunter CL, Nuwaysir LM and Schaeffer DA. The Paragon Algorithm, a next generation search

engine that uses sequence temperature values and feature probabilities to identify peptides from tandem mass spectra. Mol Cell Proteomics. 2007; 6(9):1638-1655.
